# Comparison of Transcranial Magnetic Stimulation Dosimetry between Structured and Unstructured Grids Using Different Solvers

**DOI:** 10.3390/bioengineering11070712

**Published:** 2024-07-13

**Authors:** Francesca Camera, Caterina Merla, Valerio De Santis

**Affiliations:** 1 Division of Biotechnologies, Italian National Agency for Energy, New Technologies and Sustainable Economic Development (ENEA), 00123 Rome, Italy; caterina.merla@enea.it; 2 Department of Industrial and Information Engineering and Economics, University of L’Aquila, 67100 L’Aquila, Italy; valerio.desantis@univaq.it

**Keywords:** conformal mesh, solvers comparison, structured and unstructured grids, transcranial magnetic stimulation (TMS) dosimetry

## Abstract

In recent years, the interest in transcranial magnetic stimulation (TMS) has surged, necessitating deeper understanding, development, and use of low-frequency (LF) numerical dosimetry for TMS studies. While various ad hoc dosimetric models exist, commercial software tools like SimNIBS v4.0 and Sim4Life v7.2.4 are preferred for their user-friendliness and versatility. SimNIBS utilizes unstructured tetrahedral mesh models, while Sim4Life employs voxel-based models on a structured grid, both evaluating induced electric fields using the finite element method (FEM) with different numerical solvers. Past studies primarily focused on uniform exposures and voxelized models, lacking realism. Our study compares these LF solvers across simplified and realistic anatomical models to assess their accuracy in evaluating induced electric fields. We examined three scenarios: a single-shell sphere, a sphere with an orthogonal slab, and a MRI-derived head model. The comparison revealed small discrepancies in induced electric fields, mainly in regions of low field intensity. Overall, the differences were contained (below 2% for spherical models and below 12% for the head model), showcasing the potential of computational tools in advancing exposure assessment required for TMS protocols in different bio-medical applications.

## 1. Introduction

Lately, transcranial magnetic stimulation (TMS) is undergoing significant interest due to its non-invasive approach and lower side effects than its counterparts [1,2,3,4,5,6]. TMS can be successfully used for diagnostic purposes (e.g., myelopathy, amyotrophic lateral sclerosis, and multiple sclerosis) or the treatment of several mental disorders. Recently, it received significant attention for its application in the treatment of Alzheimer’s disease in patients at the early stage of the pathology, as proposed by [7], thanks to its ability to modulate the synaptic plasticity of specific brain areas devoted to memory and cognition. In all cases, a changing magnetic field is applied to induce an electric field at a specific area of the brain through Faraday’s law of induction.

During the design stage, the use of numerical tools for predicting this induced electric field is paramount. Low frequency (LF) numerical dosimetry for TMS studies is therefore more and more relevant [8,9,10,11]. Several ad hoc (or in-house) models have been developed [12,13,14,15,16,17,18], but commercial software tools are more user-friendly and general-purpose. Among these, SimNIBS [19] and Sim4Life (ZMT, Zurich MedTech, Zürich, Switzerland) are the most widely used. The former uses a pipeline that interpolates data from a Neuroimaging Informatics Technology Initiative (NifTI) format image to obtain a model with an unstructured tetrahedral mesh, whereas the latter employs voxel-based models in a structured or rectilinear grid. The computational techniques to evaluate the induced electric fields are based on the finite element method (FEM) for both software, but with different numerical solvers. The results of these two software packages have been compared in simplified geometries consisting of a homogeneous and non-homogeneous sphere or in a realistic anatomical model of the head and brain.

In the past, several inter-comparison studies have examined LF numerical issues [20,21,22,23]. These studies employed various anatomical models, but primarily focused on uniform exposures and voxelized models prone to staircasing errors, using methods like the scalar potential finite difference (SPFD) or the scalar potential finite element (SPFE). In a study by Poljak et al. [15], different computational models and/or solvers (i.e., surface integral Equation (SIE)-based Method of Moments (SIE/MoM) [14,24], the FEM with cubical elements [12], the BEM and the hybrid FEM/BEM [25,26], and the FEM with rectilinear elements using Sim4Life software [15]) have been used for a non-uniform exposure (i.e., TMS application), but only for a homogeneous sphere or head. The only works where more realistic anatomical models for a TMS application were applied for both voxelized grids and tetrahedral mesh are found in [16,18,27]. However, the comparison in the induced electric field (E-field) was made only for a simplified homogeneous sphere in [16], or a multi-layered sphere with E-field looping tangentially on it [18,27], while for the realistic head, it was evaluated on a plane situated in the middle between the surfaces of the white and gray matter [16] to avoid evaluating the field on the boundary of tissues having different conductivities. In [27], the excitation source was also a simple circular coil far from realistic TMS applications, while in [18], the head was made of four tissues only.

In this paper, the comparison between the two solvers adopting different grids (structured and unstructured) is, instead, performed in the overall domain consisting of both homogeneous and moreover non-homogeneous spheres intentionally created to enhance the induced E-field at these discontinuities that have been poorly investigated so far. A more realistic non-homogeneous anatomical model is also considered for TMS applications (with a realistic figure-of-8 coil) to validate both commercial software. These results could sign an important advancement in exposure assessment, which is based more and more on computational tools.

## 2. Materials and Methods

### 2.1. Source Model

For this study, a typical figure-of-8 coil, the Deymed 70BF (Deymed Diagnostic, Payette, ID, USA), has been chosen as a TMS source. This coil, consisting of two adjacent circular loops with current flowing in opposite directions, produces a magnetic field with pulses waveforms approximable to a sinus at 3200 Hz. Due to the time-varying nature of the field, the E-field is induced in the underlying area of the head via the induction Faraday’s law. The focality of the induced E-field is given by the particular shape and path of the coil current, and reaches its maximum at the point of intersection between the two loops [1].

### 2.2. Computational Methods

The simulation environments chosen for this comparison are two widely available software packages: SimNIBS v.4.0.0 [19], which is based on tetrahedral mesh, and Sim4Life v7.2.4 (ZMT, Zurich MedTech, Zürich, Switzerland), which is based on a voxelized model or structured grids. All of the simulations have been run on a Workstation with a 32-Core processor at 3.69 GHz and with 256 GB RAM.

#### 2.2.1. SimNIBS

SimNIBS is an open-source pipeline for simulating the E-fields induced by TMS based on FEM and individualized head models generated from magnetic resonance images (MRIs). The pipeline automatically makes FEM tetrahedral mesh starting both from T1 + T2 MRI-scans and from only T1 MRI-scans. We used the default solver option, which consists of an algebraic multigrid preconditioned conjugate gradient solver (CG-AMG).

**Figure A1:**
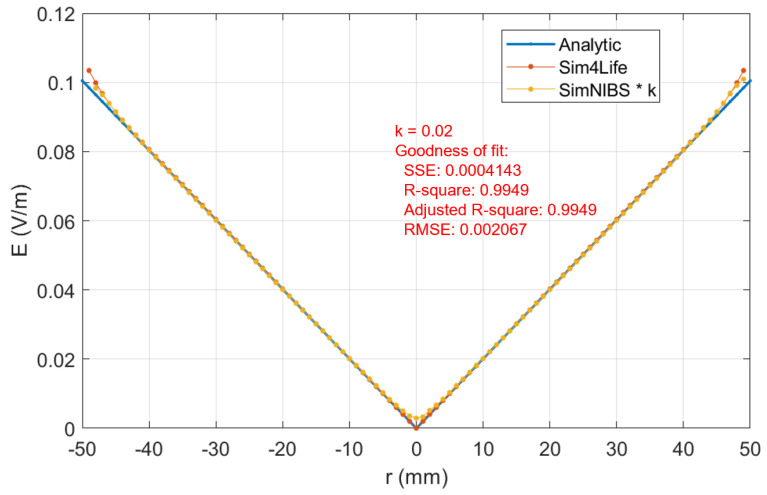
Comparison between the E-field obtained with analytical calculation (A1) and the two software packages, calculated on *y*-axis (perpendicular to B-field and passing through the center of the sphere). Blue line: E-field obtained with analytic solution (A1); red line: E-field calculated with Sim4Life; yellow line: E-field derived by multiplying SimNIBS results by a multiplicative factor k, which was determined through fitting the results to the analytical values (k = 0.02).

SimNIBS provides a built-in range of TMS coil models [28], including the Deymed 70BF. The coil shape is obtained from geometric models of the coil turns, and it is represented as a set of magnetic dipoles, from which it is possible to calculate, in a user-defined voxelized volume, the normalized magnetic induction (B-field) and the normalized magnetic vector potential (A-field) obtained when the coil is fed by a current of 1 A, using a simple formula [29]. These fields will also be used in Sim4Life to model the TMS source (see Section 2.2.2). However, SimNIBS does not allow the user to directly choose the current intensity feeding the coil (*I*), but the input for the simulations is in terms of dI/dt. Therefore, a comparison with the analytical solution using a single-shell sphere with a uniform B-field is needed to correlate the normalized B-field and the normalized A-field used as input in Sim4Life and the output of SimNIBS simulations (see Appendix A). Based on this comparison, it is obtained that the results of the simulations carried out with SimNIBS with dI/dt=1 A/μs must be divided by 50 to be compared to the ones carried out with Sim4Life (see Figure A1).

TMS simulations start by calculating the change in the A-field in the elements of the volume conductor mesh for the appropriate coil model, position and current. The time-varying A-field (dA/dt) is used as source terms to solve a linear system and obtain the electric potentials (ϕ) at the nodes of the mesh, considering the following equation:

(1) $$E=-\frac{dA}{dt}-\nabla \phi$$

#### 2.2.2. Sim4Life

Sim4Life is a simulation platform developed by the IT’IS Foundation and ZMT (Switzerland, CH) to model the interactions between physical stimuli and the human body. Similar to SimNIBS, this software can even deal with medical image data obtained from MRI. The simulation platform includes many physical solvers. Among these, the Magneto Quasi-Static (M-QS) module included in the LF Solvers (EM-LF-MQS) has been selected. This solver evaluates the induced E-field by applying the SPFE method on graded voxel grids and solving (1) in the frequency domain by setting the operation frequency to 3200 Hz.

In the EM-LF-QS solver, it is possible to add a magnetic source in two principal ways: (1) modeling the current path with dimensionless wires that replicate the coil’s windings in the desired position, or (2) importing an external source file with a user-defined discretized volume on a 3-D grid where the values of B-field and A-field are known. It should be noted that a cubic (tri-linear) interpolation is performed on the magnetic field source values from this grid (which is usually coarse) to the one used in the induced E-field evaluation (which is usually finer). Also note that if the A-field is unknown, it can be derived starting from the only knowledge of the B-field by means of the procedure described in [30], implemented in Sim4Life. In order to have a fair comparison of the field generated by the two software, the second way has been chosen, since the B-field and A-field are easily calculated in the SimNIBS coil database and exported to Sim4Life in a *.txt* file in the desired discretized volume.

Under the QS approximation, the conduction currents are at least one order of magnitude higher than the displacement currents for most of the tissues [31,32], and therefore only tissue conductivity can be considered.

### 2.3. Exposure Scenarios

**Figure 1:**
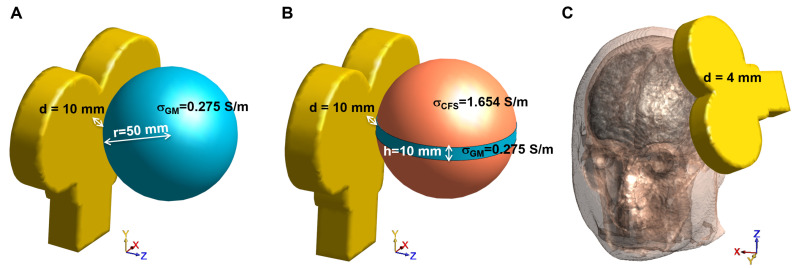
Considered exposure scenarios for the numerical comparison: (**A**) single-shell sphere, (**B**) single-shell sphere with orthogonal slab, and (**C**) MRI-derived human head model.

Three different scenarios have been considered for the numerical comparison, as shown in Figure 1.

#### 2.3.1. Single-Shell Sphere

The first scenario is a homogeneous sphere model comprised a single-shell sphere with a radius of 50 mm and conductivity of 0.275 S/m (like the gray matter GM, [33]). Preliminary simulations have been carried out varying both the voxels side of the grid (0.25, 0.5, 1 and 2 mm) and the side of tetrahedrons (see Appendix B), both for the single-shell sphere and for the single-shell sphere with slab (see Section 2.3.2). These simulations showed that the differences between the results of the two solvers are minimized when the voxels side in Sim4Life is 0.25 mm only in the homogeneous case (Table A3). This means that when a discontinuity is introduced in the model, like in Section 2.3.2 and in Section 2.3.3, the accuracy of the results is not guaranteed by shrinking the grid (Table A5).

For this reason, and also because the MRI-derived human head models are generally discretized at 1 mm, for the sake of uniformity in the comparison, in all the simulations in Sim4Life, a regular grid constituted of 1 mm side cubic voxels has been set, and the SimNIBS mesh has been chosen to have a comparable number of tetrahedrons (632,765 tetrahedrons vs. 520,613 voxels). For each simulation environment, the sphere is centered about the origin and the bottom of the coil windings is at a distance (*d*) of 10 mm above its apex at location (50, 0, 0) mm (Figure 1A).

#### 2.3.2. Single-Shell Sphere with Orthogonal Slab

The second scenario is a sphere model of the same size as the previous one, but with a conductivity of 1.654 S/m (like the cerebrospinal fluid, CSF, [33]) in which a rounded slab with 10 mm height and conductivity of 0.275 S/m (like GM) is included. As in the previous case, both the sphere and the slab are centered about the origin and the bottom of the coil windings is at a distance (*d*) of 10 mm above its apex at location (50, 0, 0) mm, as shown in Figure 1B. Once again, we handled the discretization parameters in order to obtain a number of tetrahedrons in SimNIBS comparable with that of voxels in Sim4Life with a fixed 1 mm grid (637,816 tetrahedrons vs. 520,613 voxels).

#### 2.3.3. MRI-Derived Head Model

The head model was the one provided in the SimNIBS v4.0 package Ernie, which is a human head model obtained by the segmentation *headreco* tool that segments, cleans-up the tissue maps and meshes the surfaces into triangles and volumes into tetrahedrons. The mesh consisted of nine homogeneous tissues in which the tissue conductivities were considered constant and were set as follows: σ(white matter, WM) = 0.126 S/m, σ(GM) = 0.275 S/m, σ(CSF) = 1.654 S/m, σ (scalp) = 0.465 S/m, σ(compact bone) = 0.008 S/m, σ(spongy bone) = 0.025 S/m, σ(eyeball) = 0.500 S/m, σ(blood) = 0.600 S/m, σ(muscle) = 0.160 S/m [33,34]. The coil was placed in order to mimic the dorsolateral prefrontal cortex (DLPFC) stimulation, 4 mm away from the scalp (Figure 1C). The resulting number of tetrahedrons in SimNIBS and voxels in Sim4Life (fixed 1 mm grid resolution) were 4066640 tetrahedrons vs. 5077706 voxels.

### 2.4. Metrics for Comparison

To facilitate a direct comparison of the results from the two simulation environments, the E-field calculated in SimNIBS has been interpolated for each model to derive values corresponding to the Sim4Life grid.

First, of all, a comparison of the maximum E-field obtained from each simulation ($E_{\mathrm{Max}}$ and $E_{99.9\mathrm{th}}$) has been made. $E_{99.9\mathrm{th}}$ was obtained by taking the 99.9th percentile of each GM E-field distribution, and it is, as some authors suggested [8,35,36,37,38,39], a good trade-off for localized exposures that reduces hot spots of E-field due to numerical artifacts. All of the E-field values higher than $E_{99.9\mathrm{th}}$ were considered equal to the maximum.

To further quantify the comparison, different metrics have been used. The first local metric, i.e., voxel-by-voxel, is the symmetric mean absolute percentage error (SMAPE), and it is defined as:

(2) $$SMAPE_{\mathrm{loc}}\%=100\cdot \frac{|E_{\mathrm{sim}4}-E_{\mathrm{simN}}|}{(|E_{\mathrm{sim}4}|+|E_{\mathrm{simN}}|)⁄2}$$

where $E_{\mathrm{sim}4}$ is the calculated module of the E-field with Sim4Life, while $E_{\mathrm{simN}}$ is the one calculated with SimNIBS. Note that the above expression is evaluated in each voxel, so it is possible to obtain the spatial distribution of the differences between the two solutions. If this quantity (2) is averaged on the entire domain, a global metric is obtained, i.e.,

(3) $$SMAPE\%=\frac{100}{n}\sum_{n}\frac{|E_{\mathrm{sim}4}-E_{\mathrm{simN}}|}{(|E_{\mathrm{sim}4}|+|E_{\mathrm{simN}}|)⁄2}$$

where *n* is the number of voxels of the domain in which the E-field is calculated. Other global metrics can be defined as:

(4) $$\epsilon_{1}=100\cdot \frac{\sum_{n}\left|E_{\mathrm{sim}4}-E_{\mathrm{simN}}\right|}{\sum_{n}\left|E_{\mathrm{sim}4}\right|}$$

and

(5) $$\epsilon_{2}=100\cdot \frac{\sqrt{\sum_{n}{|E_{\mathrm{sim}4}-E_{\mathrm{simN}}|}^{2}}}{\sqrt{\sum_{n}{|E_{\mathrm{sim}4}|}^{2}}}$$

These metrics consider the differences (in a global sense) between the results of SimNIBS with respect to the ones obtained in Sim4Life. Since the exposure that has been simulated in all three scenarios (Section 2.3) is a localized one, it becomes interesting to analyze the errors in the Stimulating Volume X ($SV_{X}$), which is the volume exposed to an E-field equal to or greater than X% of $E_{99.9\mathrm{th}}$.

Therefore, it is possible to define the $SMAPE{\%}_{X}$, the $\epsilon_{1X}$ and the $\epsilon_{2X}$, which are the global metrics of Equations (2)–(4) in which only the voxels where the E-field is equal to or greater than X% of $E_{99.9\mathrm{th}}$ are considered.

## 3. Results

Table 1 summarizes the maximum values of the E-field for all exposure scenarios considering an input current for the coil of 1 A. Looking at the $E_{99.9\mathrm{th}}$, the two software packages yield very close results, while the $E_{\mathrm{Max}}$ exhibits a higher discrepancy. For both software packages, $E_{\mathrm{Max}}$ overestimates the expected maximum induced field, so tetrahedral meshes are not capable of suppressing numerical errors caused by stair-casing in voxelized models when curved boundaries are approximated with voxels. However, nothing can be said about the actual maximum induced field, because, in such localized exposure with the figure-eight coil, no direct comparison with the analytic solution can be made. The contrast of conductivities between tissues and low-quality tetrahedral mesh cause numerical artifacts as well, but $E_{99.9\mathrm{th}}$ calculated for both software return stable and comparable values.

**Figure 2:**
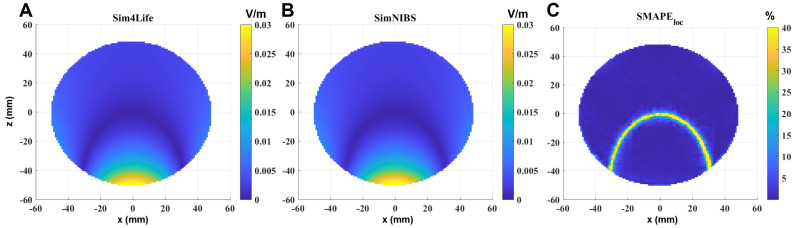
E-field induced in the sphere model on a section perpendicular to the coil and passing through the center of the sphere, calculated by (**A**) Sim4Life and (**B**) SimNIBS, and the $SMAPE_{\mathrm{loc}}$ between the two software results (**C**). Input current: 1 A. Computing time: 6 s (SimNIBS) and 9 s (Sim4Life).

An example of E-field distribution induced in a single-shell sphere model calculated in Sim4Life (panel A) and in SimNIBS (panel B) for an input current of 1 A is reported in Figure 2. The selected slice is perpendicular to the coil, and passes through the center of the sphere, i.e., also through the maximum of the localized induced E-field. By visual inspection, meshes and grids produce similar field distributions, meaning physically reasonable results. Panel C shows the $SMAPE_{\mathrm{loc}}$ calculated in the sphere. To enhance visibility, the results have been saturated to 40%. As can be observed, the error becomes significant only in the volume in which the calculated E-field is very low, so it can be given to numerical errors.

**Figure 3:**
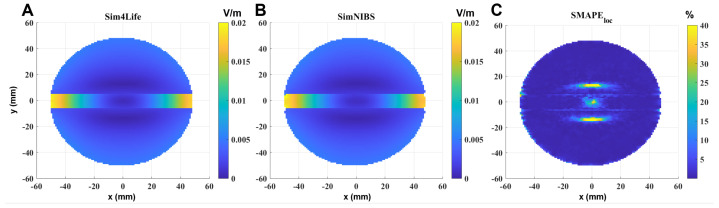
E-field induced in the sphere with orthogonal slab model on a section parallel to the coil, perpendicular to the slab and passing through the center of the sphere, calculated by (**A**) Sim4Life and (**B**) SimNIBS and the $SMAPE_{\mathrm{loc}}$ between the two software results (**C**). Input current: 1 A. Computing time: 6 s (SimNIBS) and 9 s (Sim4Life).

Similar considerations can be performed in the single-shell sphere with the orthogonal slab (Figure 3). In this case, to better visualize what happens near the discontinuity between the two materials, the selected slice is parallel to the coil, perpendicular to the slab and passing through the center of the sphere. It is also possible to see that, in this case, the error is significant and exceeds 40% only where the calculated E-field is very low, i.e., in the inner part of the sphere (due to Faraday’s law); however, the error also slightly increases near the interface between the two materials (Figure 3C).

**Figure 4:**
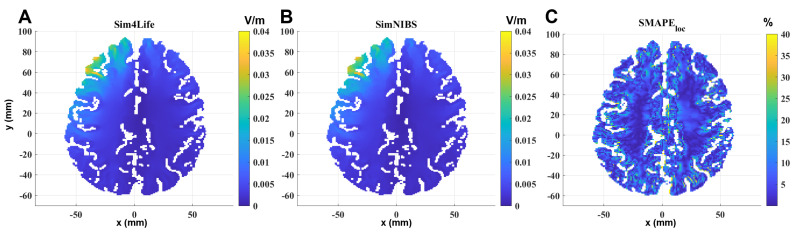
E-field induced in the MRI-derived head model in the GM and WM on a transverse section passing through the $E_{99.9\mathrm{th}}$, calculated by (**A**) Sim4Life and (**B**) SimNIBS and the $SMAPE_{\mathrm{loc}}$ between the two software results (**C**). Input current: 1 A. Computing time: 205 s (SimNIBS) and 122 s (Sim4Life).

Figure 4 shows the same comparison on a transverse section passing through the maximum of the localized induced E-field of the MRI-derived head model. Results on materials not belonging to the brain are neglected, so the calculated E-field is shown only on the GM and WM. In this case, the geometrical and electrical discontinuities are more pronounced than in the other two models, and the difference between the two solvers becomes more evident at the interface between GM and WM and between GM and materials outside the brain.

Table 2 summarizes all of the global error metrics between the two software for the three models. As the model becomes more complex, the computational differences between the two software packages increase, albeit remaining within acceptable bounds (below 5% for the spherical models and below 12% for the realistic head model).

**Figure 5:**
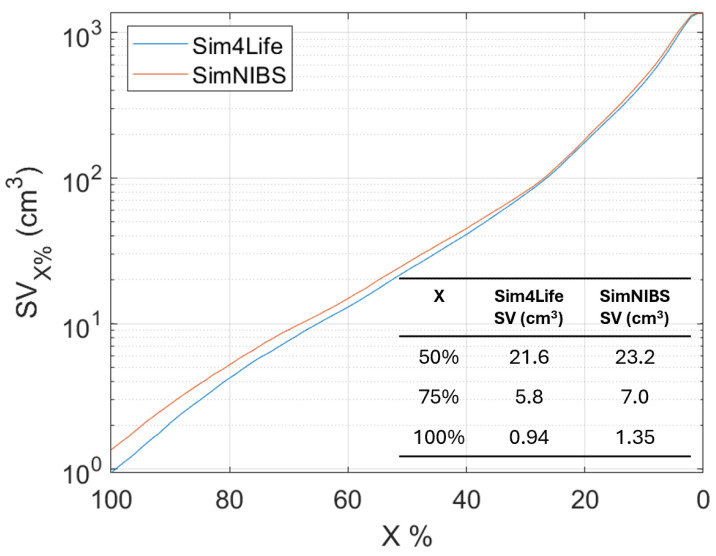
Stimulating Volume X ($SV_{X}$), i.e., the volume exposed to E-field equal to or greater than X% of $E_{99.9\mathrm{th}}$ in MRI-derived human head model varying X%.

An analysis of the Stimulating Volume X ($SV_{X}$), i.e., the volume exposed to E-field equal to or greater than X% of $E_{99.9\mathrm{th}}$, varying X% has been conducted for the head model (as shown in Figure 5). This metric is interesting when a localized exposure is considered, because it is an estimation of the focality of the stimulation. In particular, many authors [10] take the $SV_{50}$ to quantify the focality. Figure 5 shows that the focality ($SV_{50}$) slightly differs (of about 5%) between the two software.

**Figure 6:**
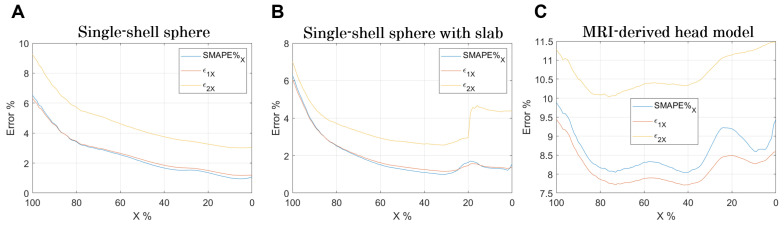
Errors in the Stimulating Volume X ($SV_{X}$), i.e., the volume exposed to E-field equal to or greater than X% of $E_{99.9\mathrm{th}}$. (**A**) Single-shell sphere, (**B**) single-shell sphere with orthogonal slab, and (**C**) MRI-derived human head model.

Finally, an analysis of the errors varying X% is shown in Figure 6. As X% decreases, the comparison is conducted over an increasingly larger volume (Figure 5). Indeed, when X=100%, it means that the comparison is made in the volume in which the E-field is equal or higher than the $E_{99.9\mathrm{th}}$, whereas when X=0%, the comparison is made in the entire volume.

Certainly, the stair-casing error, which, as already described, results in an overestimation of the E-field at certain points, and maximizes the error when the analysis focuses on that volume. However, by widening the analysis volume, the error reduces and increases again when considering volumes where the E-field is very low (Figure 6B,C).

## 4. Discussion

The comparison of LF numerical dosimetry results between conformal and non-conformal discretization of the human bodies, specifically the head and brain, has been deeply investigated [15,16,18,27]. Usually, conformal meshes are employed in solvers adopting the FEM or BEM/MoM or hybrid combinations of them for solving the induced electric field. This generally yields more accurate results, at the expense however of computationally heavier simulations. On the other hand, non-conformal solvers (e.g., SPFD or SPFE) using rectilinear grids are easier and faster to implement but suffer from staircasing errors.

When doing this discretization comparison, particular attention must therefore be paid to aligning or “matching” the nodes of the meshes with those of the grids. More precisely, the barycenter of tetrahedra should coincide with that of the voxel, in order to have a fair comparison of the dielectric properties of the material and hence of the obtained results. This is what has been made in [27,40], where special focus has been given to the parameters used to generate the meshes.

Such attention has been paid also in this study, where some parameters have been settled in SimNIBS to obtain a desired mesh. The rationale behind a fair comparison is to have a number of tetrahedral nodes similar to that of voxel numbers. This has been shown to be a good approach, as demonstrated in the supporting material with the analytic solution.

Besides paying attention to mesh generation, a lot of efforts have been made in the past to “remove” or quantify the staircasing error [8,35,36,37,38,39], finding, for instance, some smoothing techniques with fixed artifact removal (e.g., 99.9th or 99.99th [35,37,38], rather than 99th percentiles (as suggested by ICNIRP-2010 [41]) or flexible/variable removal artifact (depending on the grid resolution employed) [36,39]. However, among these suggested approaches, no one can be perfect in removing 100% of the numerical artifacts, as no analytic or experimental solution exists for realistic anatomical models. This is erroneously stated in [16], even though we can draw the same conclusions that choosing a grid resolution of 1 mm (or below) is retained to be good enough for structured or rectilinear grids. Once again, this is confirmed by the results shown in our and their supporting materials when comparing the induced E-field in a homogeneous sphere with the analytic solution. Further, looking at the errors reported in Figure 6, it emerges that their minimization is for X% between 40% and 60% for both software. This result indicates this volume % range as the optimal one for dosimetric assessment.

The comparison between structured and unstructured numerical solvers revealed that errors become more pronounced with increasing geometric complexity, this finding has several practical implications for TMS applications.

This comparison could be helpful when it is required to target small and specific brain regions. This is of interest for TMS applications in treating conditions such as Alzheimer’s disease [7]. Their approach focuses on stimulating specific areas of the brain, which requires precise modeling to ensure effectiveness of the treatment. The increased error in complex geometries indicates the need for advanced solvers to accurately define exposure also in interconnected brain areas. This is important for TMS applications that aim to target networks of brain regions. Accurate modeling can help in understanding the effects of TMS on these interconnected areas, leading to more effective treatments. Understanding the limitations and potential errors of different numerical solvers can aid in optimizing TMS protocols. Clinicians and researchers can choose the most appropriate solver based on the complexity of the target geometry, improving the precision of TMS treatments and potentially enhancing clinical outcomes. In summary, the results underscore the importance of selecting the right numerical solver for accurate TMS application, particularly when dealing with complex brain geometries. This has direct implications for the treatment of neurological conditions like Alzheimer’s disease [7], as well as for the broader use of TMS in targeting specific and interconnected brain areas.

Finally, regarding the comparison between the two commercial software packages, no preferences or endorsements are made. The computation time for the two software packages is comparable (below 10 s for the two spherical model, and about 2–3 min for the head model, cfr. Figure 2, Figure 3 and Figure 4). The choice between the software packages should be based on practical considerations, such as the type of sources available. SimNIBS, for example, offers more specific commercial coils built into the software, while the other software may require the realization of specific geometries. Sim4Life, for instance, includes permittivity values for tissues, which are not included by default in the other software, and could be relevant in specific applications.

The flexibility in terms of GUI and computing interface varies, and the operator may prefer one over the other based on their skill set. Both software packages are user-friendly for standard cases and deliver computational results quickly.

Overall, these tools have the potential to open numerous doors in the treatment of various mental disorders using TMS applications. They allow for rigorous assessment of E-field values for different protocols in real-time patient scenarios.

## 5. Conclusions

In this study, a comparison of TMS dosimetry between structured and unstructured grids using different solvers was performed with the two most commonly used software packages for TMS dosimetry: Sim4Life and SimNIBS. The comparison was conducted on three different geometrical models of increasing complexity: a homogeneous sphere, a sphere with an internal discontinuity, and a head model derived from MRI data.

The results demonstrate that differences between the obtained results are larger as geometric complexity increases. However, these differences remain on overall contained (below 5% for spherical geometric models and below 12% for the head model) and locally significant only in areas of tissues where the electric field value is very low (and therefore much more susceptible to numerical errors) or, as expected, near the tissues discontinuities. These results could sign an important advancement in exposure assessment protocols, which are based more and more on computational tools.

## Figures and Tables

**Table 1:** Maximum value of E-field for the three exposure scenarios calculated by both software with an input current of 1 A. ($E_{\mathrm{Max}}$) is the maximum E-field obtained from each simulation and $E_{99.9\mathrm{th}}$ was obtained by taking the 99.9th percentile of each GM E-field distribution.

	Sim4Life		SimNIBS	
	**Max**	**99.9th**	**Max**	**99.9th**
	**(mV/m)**	**(mV/m)**	**(mV/m)**	**(mV/m)**
Single-shell sphere	38.3	29.1	34.0	29.1
Single-shell sphere with slab	82.6	79.0	100.1	78.3
MRI-derived head model	43.0	27.9	38.0	27.2

**Table 2:** Global error metrics between the results of the two software for the three dosimetric models.

	SMAPE%	$\epsilon_{1}$	$\epsilon_{2}$
Single-shell sphere	1.1	1.2	3.0
Single-shell sphere with slab	1.6	1.4	4.4
MRI-derived head model	9.4	8.6	11.5

## Data Availability

Data are available from the corresponding author upon reasonable request.

## Appendix A

Since in SimNIBS the magnetic source can be generated only in the form of magnetic dipoles, in the case of the spatially uniform exposure, the magnetic vector potential A was given by a strong point-wise magnetic dipole placed very far away from the head, in order to obtain a spatially uniform B-field at 3200 Hz of 0.2 mT, directed along the *x*-axis. The spatial distribution of the induced E-field in a homogeneous conductive sphere is well-known, i.e., the analytic solution exists and is given by:

(A1) $$E=\frac{\omega Br}{2}=\pi fBr$$

where *f* = 3200 Hz, *B* = 0.2 mT and *r* is the radial direction.

Figure A1 shows the magnitude of the E-field along this radial direction. This quantity is calculated with Equation (A1) (blue line) and with Sim4Life (red line). The yellow line is the E-field derived by multiplying SimNIBS results by a multiplicative factor k, which was determined through fitting the results to the analytical values (found to be equal to 0.02).

## Appendix B

In this appendix, a comparison between several meshes and grid discretizations will be provided in order to compare the results obtained in SimNIBS and Sim4Life.

Five different tetrahedral single-shell sphere models were generated with different resolutions in SimNIBS, as reported in Table A1, whereas four different voxels side of the grid have been chosen for simulations in Sim4Life (see Table A2).

**Table A1:** Tetrahedral meshes for single-shell sphere models (SimNIBS).

	Mean Edge Length (mm)	Tetrahedra ($\times 10^{6}$)	Nodes ($\times 10^{6}$)
Mesh 1	2.64	0.30	0.05
Mesh 2	2.03	0.63	0.11
Mesh 3	1.20	3.07	0.52
Mesh 4	0.80	9.46	1.56
Mesh 5	0.54	30.90	5.07

**Table A2:** Number of voxels in different grids for single-shell sphere models (Sim4Life).

	Voxels Side (mm)	Voxels ($\times 10^{6}$)
Grid 1	2.00	0.07
Grid 2	1.00	0.52
Grid 3	0.50	4.17
Grid 4	0.25	33.32

In Table A3, the *SMAPE%* obtained by comparing all the combinations between different meshes and different voxel sizes is reported. Decreasing the mean tetrahedral edge length from Mesh 3 to Mesh 4 and Mesh 5 does not significantly reduce the errors with Sim4Life. On the contrary, decreasing the grid size in Sim4Life always reduces the errors with SimNIBS. However, a convergence trend is almost found at around (or less) 1 mm grid size.

**Table A3:** *SMAPE%* between the results of different meshes/grid sizes for single-shell sphere.

	Grid 1	Grid 2	Grid 3	Grid 4
Mesh 1	1.54	1.17	1.00	0.93
Mesh 2	1.44	1.08	0.90	0.83
Mesh 3	1.38	0.97	0.76	0.67
Mesh 4	1.37	0.95	0.75	0.66
Mesh 5	1.38	0.95	0.75	0.66

For the single-shell sphere with slab, only two different tetrahedral meshes have been tested in SimNIBS (Mesh 6 and Mesh 7, which have similar characteristics to Mesh 2 and Mesh 3; see Table A3 and Table A4). In Table A5, the *SMAPE%* obtained comparing all the combinations between different meshes and different voxel sizes is reported. It is possible to notice that minimizing the grid size to 0.25 mm does not guarantee the error minimization. This is probably due to the local enhancement of the induced E-field around the low-lossy slide.

**Table A4:** Tetrahedral meshes for single-shell sphere model with slab (SimNIBS).

	Mean Edge Length (mm)	Tetrahedra ($\times 10^{6}$)	Nodes ($\times 10^{6}$)
Mesh 6	2.03	0.64	0.11
Mesh 7	1.20	3.08	0.52

**Table A5:** *SMAPE%* between the results of different meshes/grid sizes for single-shell sphere with slab.

	Grid 1	Grid 2	Grid 3	Grid 4
Mesh 6	2.30	1.56	2.33	2.45
Mesh 7	1.89	1.00	1.98	2.11
